# Public reporting of IVF outcomes influences medical decision-making and physician training

**DOI:** 10.1186/s40738-020-00070-7

**Published:** 2020-02-11

**Authors:** Stephanie Gunderson, Emily S. Jungheim, Caleb B. Kallen, Kenan Omurtag

**Affiliations:** 1 Department of Obstetrics and Gynecology at Washington University Division of Reproductive Endocrinology St Louis, St. Louis, MO USA; 2 Department of Obstetrics and Gynecology at Northwestern University Division of Reproductive Endocrinology Chicago, Chicago, IL USA; 3 Shady Grove Fertility Philadelphia, Philadelphia, PA USA

**Keywords:** SART reporting, Public reporting, CDC reporting, IVF outcomes, Survey

## Abstract

**Background:**

Since 1992 ART clinics have been required to report outcome data. Our objective was to assess practitioners’ opinions of the impact of public reporting of assisted reproductive technology (ART) outcomes on treatment strategies, medical decision-making, and fellow training.

**Methods:**

Survey study performed in an academic medical center. Members of the Society of Reproductive Endocrinology and Infertility and the Society of Reproductive Surgery were recruited to participate in an online survey in April 2012.*:* Categorical survey responses were expressed as percentages. Written responses were categorized according to common themes regarding effects of reporting on participants’ medical management of patients. The study was primarily qualitative and was not powered to make statistical conclusions.

**Results:**

Of 1019 surveys sent, 323 participants (31.7%) responded from around the United States, and 275 provided complete data. Nearly all (273 of 282; 96.8%) participants responded that public reporting sometimes or always affected other providers’ practices, and 264 of 281 (93.9%) responded that other practitioners were motivated to deny care to poor-prognosis patients to improve reported success rates. However, only 121 of 282 (42.9%) indicated that public reporting influenced their own medical management. The majority of respondents agreed that public reporting may hinder adoption of single embryo transfer practices (194 of 299; 64.9%) and contribute to the persistent rate of twinning in in vitro fertilization (187 of 279; 67%). A small majority (153 of 279; 54.8%) felt that public reporting did not benefit fellow training, and 58 (61.7%) of the 94 participants who trained fellows believed that having fellows perform embryo transfers reduced pregnancy rates. A small majority (163 of 277; 58.8%) of respondents reported their ART success rates on clinical websites. However, the majority (200 of 275; 72.7%) of respondents compared their success rates with those of other clinics. Finally, most respondents (211 of 277; 76%) believed that most centers that advertised their success rates did so in ways that were misleading to patients.

**Conclusions:**

Public reporting of ART clinical outcomes is intended to drive improvement, promote trust between patients and providers, and inform consumers and payers. However, providers reported that they modified their practices, felt others denied care to poor-prognosis patients, and limited participation of trainees in procedures in response to public reporting of ART outcomes.

## Introduction

In 1985, shortly after the introduction of in vitro fertilization (IVF) in the United States, the Society of Assisted Reproductive Technology (SART) was established and began collecting data from its clinic members [1, 2]. In 1992, Congress responded to consumer complaints regarding the lack of available clinical success rate data from assisted reproductive technology (ART) practices and the article “Are we exploiting the infertile couple?” by passing the Fertility Clinic Success Rate and Certification Act [3–5]. This law mandated the reporting of ART success rates to the Centers for Disease Control (CDC) [6]. Reporting of ART outcomes, in theory, empowers prospective and current patients to make informed decisions about their treatment and choice of practitioners by providing accurate and transparent outcomes data. In addition, reporting is intended to motivate fertility centers to practice high-quality, evidence-based care. In 2015, 499 (93%) of fertility centers offering ART services reported clinical outcomes to the CDC [7].

The benefits of public reporting of ART outcomes may be mitigated by unintended consequences of public reporting. First, third parties use publicly reported data to publish “rankings” of IVF centers that are based upon ART success rates. However, these rankings do not consistently consider important variables such as patient demographics or adverse clinical outcomes such as higher-order multiples and ovarian hyperstimulation syndrome [8–11]. Second, clinics often advertise their success rates on websites, in conflict, perhaps, with society guidelines intended to promote fair and meaningful data sharing without undue bias or deception [12]. Finally, because competition between centers exists, providers may be driven to perform in ways, that may be interpreted by some, to not entirely benefit their patients (e.g., by taking unnecessary risks or denying care to poor-prognosis patients) or trainees (e.g., by limiting trainee involvement in ART procedures).

Our study aimed to address this last concern by surveying ART providers about how public reporting affects medical decision-making and the clinical training of future ART providers.

## Materials and methods

### Study design

This was a cross-sectionalsurvey conducted in April 2012 of reproductive endocrinology and infertility clinicians who were members of the Society of Reproductive Endocrinology and Infertility or the Society of Reproductive Surgery as described previously [13]. Briefly, 1019 physicians were sent, via email, electronic surveys (Additional file 2). Weekly reminders were sent out for 4 weeks, and survey responses were collected over 1 month. This study was approved by the Institutional Review Board at Washington University, St Louis.

### Statistical analysis

All survey data were analyzed, including incomplete surveys. Categorical survey responses are expressed as percentages; where appropriate, means and standard deviations are reported. Written responses were reviewed and categorized according to several common themes. The study was primarily qualitative and not powered to make statistical conclusions.

## Results

Three hundred twenty-three members (31.7% response rate) of the Society of Reproductive Endocrinology and Infertility or the Society of Reproductive Surgery answered online surveys, and 275 provided responses to all questions that were applicable (only 94 of the clinicians surveyed train clinical fellows). Table 1 shows practitioner characteristics including age, gender, years in practice, type of practice, and number of IVF cycles performed per year. More participants were male than female, and more participants categorized their practices as private than academic/public or academic/private. All regions of the country were represented (Additional file 1: Table S1).

**Table 1:** Demographics of 312 Survey Respondents

Gender	N
Male	195 (62.5%)
Female	117 (37.5%)
**Practice Setting**	
Private Practice	178 (57.1%)
Academic/Public	55 (17.6%)
Academic/Private	72 (23.1%)
Other	7 (2.2%)
**Age (mean, SD)**	49.7 (9.5)
**IVF Cycles/yr (median, range)**	300 (20–5000)
Yrs in Practice (mean, SD)	16.7 (10.3)

Nearly all (273 of 282; 96.8%) respondents believed that public reporting affected other providers’ practices, and 264 of 281 (93.9%) believed that other providers denied IVF to poor-prognosis patients to improve reported success rates (Table 2). In contrast, only 121 of 282 (42.9%) of respondents indicated that their own medical management was influenced by public reporting of IVF outcomes. When providers were asked to elaborate as to how public reporting influenced their practices 16.7% reported they decreased the number of embryos transferred while 22.8% reported that they increased the number of embryos transferred. Additionally, when asked directly,only 78 of 281 (27.8%) believed that public reporting influenced their own willingness to offer non-donor, fresh single-embryo transfer (Table 2).

**Table 2:** Perceived/Reported Effects of Public Reporting on Medical Decision Making, N (%)

Question	Always	Sometimes	Never	Not Sure
Reporting influences OTHER’s practice	67 (23.8)	206 (73)	3 (1.1)	6 (2.1)
Reporting motivates OTHER’s to deny IVF to poor prognosis patients in order to improve reported success rates	47 (16.7)	217 (77.2)	1 (0.4)	16 (5.7)
Question	**YES**		**NO**	
Reporting influences YOUR willingness to offer non-donor, fresh eSET	78 (27.8)		203 (72.2)	
Reporting influences YOUR medical management	121 (42.9)		161 (57.1)	
If so how?	114 written responses reviewed			
Decrease number of embryos transferred	19 (16.7)			
Increase number of embryos transferred	26 (22.8)			
Deny care to poor prognosis patients	44 (38.6)			
Other	25 (21.9)			

Regarding benefits of public reporting of IVF outcomes (Table 3), only 49 of 278 (17.6%) respondents believed that reporting benefits education and training of fellows. Nonetheless, a majority of respondents (165 of 279; 59.1%) believed that couples seeking infertility treatment benefit from public reporting of IVF outcomes. A minority of respondents believed that public outcomes reporting drives scientific advancements in the field (123 of 279; 44.1%) and promotes quality improvements (130 of 279; 46.6%). Specifically, 189 of 274 (69%) respondents believed that public reporting hinders the adoption of elective SET and 67% believed that public reporting contributed to the persistent rate of twinning in IVF. Finally, a minority of respondents believed that public reporting benefits patient satisfaction (62 of 279; 22.2%) and information sharing among groups (75 of 279; 26.9%) (Table 3). A small majority (163 of 277; 58.8%) of respondents advertise their success rates on clinical websites. However, most respondents compared their success rates with other clinics websites (200 of 275; 72.7%), and most (211 of 277; 76.2%) believed that clinical advertising was misleading to patients. The majority (85 of 94; 90.4%) of respondents who train clinical fellows believed that fellows did not reduce a center’s pregnancy rates by performing oocyte retrievals, but 58 of 94 (61.7%) believed that fellows reduce a center’s pregnancy rates by performing embryo transfers (Table 4).

**Table 3:** Public Reporting Influence. N (%)

Question	YES	NO	Not Sure
Couples seeking infertility treatment	165 (59.1)	78 (28)	36 (12.9)
Fellow training/education	49 (17.6)	153 (54.8)	77 (27.6)
Clinicians	110 (39.4)	110 (39.4)	59 (21.1)
Advancements in the field	123 (44.1)	106 (38)	50 (17.9)
ART twinning rate	183 (66.8)	47 (17.2)	44 (16.1)
Adoption of eSET	189 (69)	47 (17.2)	38 (13.9)
Improved quality initiatives in infertility care	130 (46.6)	124 (44.4)	25 (9)
Improved treatment protocols	108 (38.7)	142 (50.9)	29 (10.4)
Improved patient satisfaction	62 (22.2)	164 (58.8)	53 (19)
Information sharing among groups	75 (26.9)	161 (57.7)	43 (15.4)

**Table 4:** On the Influence of Public Reporting on Fellow Training, N (%)

Question	YES	NO
Oocyte retrievals by fellows harms pregnancy rates	7 (7.7)	84 (92.3)
Embryo transfers by fellows harms pregnancy rates	56 (61.5)	35 (38.5)
Does public reporting influence how fellows are trained	10 (11.0)	81 (89.0)

## Discussion

Public reporting of IVF outcomes is based on the premise that data transparency enhances provider accountability and encourages improvements in clinical care and outcomes. However, our study suggests that public reporting has two important unintended consequences for patients. First, IVF clinicians may deny care to poor-prognosis patients due to concerns of poor response, increase in cancelled cycles and lower live birth rates as a result of higher rates of aneuploidy. This is especially problematic given that more and more women in the United States have been deferring childbearing to later years. Second, at the time of this survey, providers may transfer more than the recommended number of embryos in an attempt to improve their clinics’ success rates. In the most recent update of Assisted Reproductive Technology Surveillance – United States 2015, 34.7% of women under the age of 35 received a single embryo, 34% of babies born via ART were twins, and 1% were triplets [7]. In a survey study published in 2010, 94% of respondents reported routinely using ASRM guidelines for embryo transfers, but 52% would deviate from guidelines at patient request [14]. Although respondents in that study reported routinely discussing risk of multiple gestations with their patients, only 34% discussed the merits of single-embryo transfer [14]. We recognize that both the survey study from 2010 as well as our study are 8+ years old and that elective single embryo transfer is now not elective but rather the standard of care in many clinics around the world. The ability to better select the single best embryo to transfer using PGT as well as other clinical and morphological parameters has increased clinicians’ confidence in transferring a single embryo without harming clinic success rates. As a consequence of increasing the single embryo transfer rate, the multiple birth rate resulting from ART as decreased dramatically over the last decade [15]. It is also worth noting that our survey findings precede efforts by the CDC and SART to update outcome reporting to help dissuade patient selection and avoidance of single embryo transfer.

Public reporting may also have unintended consequences for trainees, leading practices to not allow fellows to perform live embryo transfers for fear of reducing a center’s success rates. However, these fears seem to be unwarranted, as two studies found no difference in live birth rates between embryo transfers performed by fellows and those performed by attendings [16, 17]. Additionally, the use of embryo transfer simulators improved fellows’ live birth rates with their first 10 live transfers [18]. One concern of allowing fellows to perform embryo transfers is that their 18 months of dedicated research time could reduce their success rates, but at least one study showed that this was not the case [19].

In some respects, our findings mirror those in the field of interventional cardiology. This field has publicly reported observed and risk-adjusted mortality rates since 1994. In a 2005 survey of interventional cardiologists, 79% of respondents agreed or strongly agreed that the publication of individual mortalities influenced their decision making, and 83% believed to some extent that patients who may benefit from angioplasty were not treated due to this reporting [20]. Thus, our field is not alone in grappling with the potential unintended consequences of public reporting of outcomes.

Reporting IVF outcomes is challenging for two main reasons. First, reporting can include multiple numerators (e.g., pregnancy rate, ultrasound-documented pregnancy rate, and live birth rate) and denominators (e.g., per initiated cycle, per retrieval, and per embryo transfer), making it difficult for patients to interpret reported ART success rates [21, 22]. Although the American Society for Reproductive Medicine [23] and the American Medical Association published guidelines to address concerns regarding inaccuracies and incomplete information provided on public health websites [24], many clinics fail to adhere to these guiding principles [23–27].

In response to the lack of uniformity among ART clinical website reporting SART states “a comparison of clinic success rates may not be meaningful because patient medical characteristics and treatment approaches may vary from clinic to clinic” [1]. Interestingly, ART practices in Spain differ from that of the US and are unique in that there are both mandatory and voluntary ART registries. In 2010, Luceno et al. compared the reporting rates in both the mandatory and voluntary registries in Spain and noted that the voluntary data were valid in fresh, nondonor cycles [28]. Of the respondents in our study, 114 of 277 (41.2%) did not report their success rates on clinical websites. However, many respondents did compare their success rates with other clinics (200 of 277 (72.7%)) and believed that clinical website reporting of success rates was misleading (211 of 277 (76.2%)).

Second, reporting guidelines always lag continuously behind evolving ART practices. For example, as oocyte and egg vitrification has improved, and the numbers of oocyte and embryo banking cycles have increased, the reported success rates (defined as the number of cycles with reported pregnancies divided by the total number of initiated cycles) have decreased [29, 30]. Additionally, the advent of preimplantation genetic testing for aneuploidy or for the diagnosis of single gene disorders has increased the number of IVF cycles with delayed/cancelled embryo transfers, which similarly reduces reported IVF success rates per initiated cycle [31–33].

This is the first study to evaluate provider perceptions of the effects of IVF outcomes reporting. Although our survey had a high response rate, our study has several limitations. First, physicians’ responses may have been influenced by their preexisting biases regarding SART/CDC reporting of IVF outcomes. The majority of our respondents (57.1%) identified their practice setting as private. We recognize that there are differences in practice patterns dependent on practice setting that could also bias our study findings. Second, the views of the non-respondents may have differed from those of the responders. Third, our survey was conducted in 2012, and the ART field has changed substantially in the last 7 years. Most notably, the uses of preimplantation genetic testing and diagnosis as well as embryo vitrification have increased, leading to a decrease in the rate of multiple gestations as a consequence of an increase in the single embryo transfer rate [15]. Additionally, embryo transfer simulation has been introduced into training for fellows.

## Conclusions

These data provide a very important starting point for assessing provider perceptions about public reporting. We believe it is important to understand how public reporting influences decisions of ART providers, especially regarding the utilization of single embryo transfers as well as fellow involvement in procedures. Future work should be focused on addressing questions such as whether respondents still believe that public reporting influences fellowship training considering the increasing use of embryo transfer simulators. Also, it is likely that payors will continue to use federally mandated data points to identify, so called “Centers of Excellence,” a behavior that might push more clinics to perform preimplantation genetic testing for aneuploidy to keep up with local market forces while still encouraging single embryo transfers.

## Additional files

**Additional file 1: Table S1.** Geographic Distribution of 73% of 312 Respondents, N (%).

**Additional file 2:** Physician survey regarding public reporting of In Vitro Fertilization (IVF) outcomes by SART/CDC.

## Data Availability

The datasets during and/or analyzed during the current study available from the corresponding author on reasonable request.
