# Supplementary material for: Public reporting of IVF outcomes influences medical decision-making and physician training
Source: Fertil Res Pract. 2020 Feb 11;6:1. doi: 10.1186/s40738-020-00070-7 (PMC7014742; doi:10.1186/s40738-020-00070-7)
Supplement: Supplementary file 1 — Additional file 1: Table S1. Geographic Distribution of 73% of 312 Respondents, N (%). [file 40738_2020_70_MOESM1_ESM.docx]

| SUPPLEMENTAL TABLE 1 Geographic Distribution of 73% of 312 Respondents, N (%) | |
| --- | --- |
| California | 39 (12.5) |
| New York | 20 (6.4) |
| Texas | 20 (6.4) |
| Pennsylvania | 19 (6.1) |
| Florida | 18 (5.8) |
| Maryland | 16 (5.1) |
| Michigan | 16 (5.1) |
| Massachusetts | 13 (4.2) |
| Illinois | 12 (3.8) |
| North Carolina | 12 (3.8) |
| Virginia | 10 (3.2) |
| Colorado | 10 (3.2) |
| New Jersey | 9 (2.9) |
| Georgia | 8 (2.5) |
| Missouri | 8 (2.5) |
