# Supplementary material for: Public reporting of IVF outcomes influences medical decision-making and physician training
Source: Fertil Res Pract. 2020 Feb 11;6:1. doi: 10.1186/s40738-020-00070-7 (PMC7014742; doi:10.1186/s40738-020-00070-7)
Supplement: Supplementary file 2 — Additional file 2: Physician survey regarding public reporting of In Vitro Fertilization (IVF) outcomes by SART/CDC. [file 40738_2020_70_MOESM2_ESM.docx]

**Title: Physician survey regarding public reporting of In Vitro Fertilization (IVF) outcomes by SART/CDC.**

**For whom is this survey intended?**

**Answer:** Physicians who perform IVF. If you are not a physician who treats infertile couples, please disregard this questionnaire.

**What are you being asked to do?**

**Answer:** We are asking you to *voluntarily* complete a brief, anonymous survey that takes about 5 minutes to complete.

**Why are we asking you to do this?**

**Answer:** This survey is part of a research study to explore the unintended consequences of public reporting of IVF outcomes by SART/CDC.

**Any questions?**

- For questions, please contact Kenan Omurtag MD (314-286-2400). This questionnaire is IRB approved and if you wish to speak with someone else, or if you have questions or concerns regarding your rights as a research subject, call Dr. Jonathan Green MD, Chairman of the University’s Human Research Protection Office, at (314) 633-7400 or (800) 438-0445.

Respondent Demographics:

1. Describe your clinical practice:
   1. Private practice
   2. Private University based/affiliated practice
   3. Public University based/affiliated practice
   4. Other, please specify:_______________________________
2. In what state do you primarily practice? (Toggle Here: 50 States)

Public Reporting and Training Clinical Fellows:

1. Do you train clinical fellows in Reproductive Endocrinology and Infertility?
   1. Yes
   2. No

If you answered "NO" to this question, skip to question #10:

1. Do clinical fellows perform **oocyte retrievals** in your center?

Never---------Sometimes--------Always

1----------2----------3----------4-----------5

1. Do you worry that clinical fellows performing retrievals might harm your reported pregnancy rates?

Never---------Sometimes--------Always

1----------2----------3----------4-----------5

1. Do clinical fellows routinely perform **embryo transfers**?

Never---------Sometimes--------Always

1----------2----------3----------4-----------5

1. Do you worry that clinical fellows performing embryo transfers might harm your reported pregnancy rates?

Never---------Sometimes--------Always

1----------2----------3----------4-----------5

1. Do you worry that graduating fellows may become your competitors in the local/regional medical marketplace?

Never---------Sometimes--------Always

1----------2----------3----------4-----------5

1. Has public reporting of IVF outcomes influenced how you train your fellows (i.e. whether you share all of your "clinical pearls" with your trainees)?

Never---------Sometimes--------Always

1----------2----------3----------4-----------5

Public Reporting and Medical Decision Making:

1. Has public reporting of IVF outcomes ever influenced your clinical practice patterns/medical decision making?

Never---------Sometimes--------Always

1----------2----------3----------4-----------5

1. Have you ever offered IVF to good prognosis patients (even when less aggressive therapies might succeed) in order to maintain optimal reported pregnancy rates?

Never---------Sometimes--------Always

1----------2----------3----------4-----------5

1. Have you ever managed the IVF cycles of good prognosis patients more aggressively than you ordinarily would (i.e. stimulated harder, transferred more embryos) in order to maintain optimal reported pregnancy rates?

Never---------Sometimes--------Always

1----------2----------3----------4-----------5

1. Have you ever denied poor-prognosis patients IVF therapy in order to maintain optimal reported pregnancy rates?

Never---------Sometimes--------Always

1----------2----------3----------4-----------5

1. Do you believe that public reporting of IVF outcomes influences the clinical practice patterns or medical decision making of **other providers**?

Never---------Sometimes--------Always I'm not sure

1----------2----------3----------4-----------5

1. Do you believe that some providers favor IVF for patients who might not need IVF in order to boost their reported pregnancy rates?

Never---------Sometimes--------Always I'm not sure

1----------2----------3----------4-----------5

1. Do you believe that some providers deny IVF to poor-prognosis patients in order to maintain optimal reported pregnancy rates?

Never---------Sometimes--------Always I'm not sure

1----------2----------3----------4-----------5

1. Does your practice employ ovarian reserve thresholds below which IVF is **not** offered (i.e. do you deny IVF therapy to women who test below a stated or unstated threshold of ovarian reserve)?

Never---------Sometimes--------Always

1----------2----------3----------4-----------5

1. If women with diminished ovarian reserve are denied IVF therapy, is this so that your center can maintain optimal reported pregnancy rates?

Never---------Sometimes--------Always or Not Applicable

1----------2----------3----------4-----------5

Public Reporting and Improvement of Patient Care:

1. Do you believe that, in aggregate, public reporting of IVF outcomes has **benefitted couples** seeking fertility treatments?
   1. Yes
   2. No
   3. I'm not sure
2. Do you believe that, in aggregate, public reporting of IVF outcomes has **benefitted the process of training fellows** in Reproductive Endocrinology and Infertility?
   1. Yes
   2. No
   3. I'm not sure
3. Do you believe that, in aggregate, public reporting of IVF outcomes has **benefitted the clinicians** who provide fertility services?
   1. Yes
   2. No
   3. I'm not sure
4. Do you believe that, in aggregate, public reporting of IVF outcomes has **promoted advancements in the field of fertility care**?
   1. Yes
   2. No
   3. I'm not sure
5. Do you believe that public reporting of IVF outcomes has **promoted improved quality initiatives in your center**?
   1. Yes
   2. No
   3. I'm not sure
6. Do you believe that public reporting of IVF outcomes has **promoted improved treatment protocols in your center**?
   1. Yes
   2. No
   3. I'm not sure
7. Do you believe that public reporting of IVF outcomes has **promoted technological advancements in your center**?
   1. Yes
   2. No
   3. I'm not sure
8. Do you believe that public reporting of IVF outcomes has **improved patient satisfaction in your center**?
   1. Yes
   2. No
   3. I'm not sure
9. Do you believe that, in aggregate, public reporting of IVF outcomes has **promoted information sharing between providers in the field of fertility care**?
   1. Yes
   2. No
   3. I'm not sure

Website-related Questions:

1. Does your center report the outcomes of IVF cycles on a proprietary website (i.e. independent of the SART/CDC websites)?
   1. Yes
   2. No
2. Have you ever compared your center's reported IVF success rates to those of other fertility practices?
   1. Yes
   2. No
3. If you have looked at **proprietary websites** of other fertility centers, do you believe that some centers present their IVF success rates in ways that are misleading to prospective patients?
   1. Yes
   2. No
   3. I'm not sure
   4. Not applicable

Possible space for people to enter personal comments regarding public reporting of IVF outcomes by SART/CDC: _______________________
